# A Medical Psychologist of the Seventeenth Century

**Published:** 1860-07-01

**Authors:** 


### Art. II.—A MEDICAL PSYCHOLOGIST OF THE SEVENTEENTH CENTURY.

We wish to introduce to our readers a worthy of the seventeenth century. John Bulwer, to wit. He does not take rank with the great ones of his day, and yet he well deserves to be had in remembrance. He would claim this of us from his writings alone; first, on account of their intrinsic interest, and, secondly, from the illustration they afford of the early effects of Bacon's method and teachings. But a higher claim may be advanced, inasmuch as Bulwer was the first Englishman who systematically gave himself up to the search for a scheme by which the moral and intellectual cultivation of deaf-mutes might be effected.

Of himself we know little or nothing, except what is derived from his works, and we cannot even say whether these, dated from 1644 to 1653, were published in the prime or the decline of his life. He was a physician, and the son of a physician, and he constituted a portion of the better mental soil which existed at that period, of which Macaulay has thus written:—

> "While the lighter literature of England . . . . was becoming a nuisance and a national disgrace, the English genius was effecting in science a revolution which will, to the end of time, be reckoned among the highest achievements of the human intellect. Bacon had sown the good seed in a sluggish soil and an ungenial season. He had not expected an early crop, and his last testament had solemnly bequeathed his fame to the next age. During a whole generation his philosophy had, amidst tumults, wars, and proscriptions, been slowly ripening in a few well-constituted minds. While factions were struggling for dominion over each other, a small body of sages had turned away with benevolent disdain from the conflict, and had devoted themselves to the nobler work of extending the dominion of man over matter. As soon as tranquillity was restored, these teachers easily found attentive audiences. For the discipline through which the nation had passed had brought the public mind to a temper well fitted for the reception of the Verulamian doctrine."*

Among the small body of sages thus referred to, Bulwer deserves to hold a note-worthy place. True, his works abound with those credulous eccentricities which too frequently disfigure the writings of his day on experimental philosophy (then but merely dawning) and which were made the butt of Butler's bitter satire. True, that Bulwer is, perhaps, best known at the present time, among bookworms, by the curious and wonderful recitals which are found in his last and most popular work; yet notwithstanding these things, we think it may be shown that his works form a happy illustration of the early ripening of Bacon's method, and that thus they may be regarded as an apt, if an odd comment upon the sentences we have just quoted from Macaulay. Further, Bulwer's writings have a special interest for us, because they contain an early attempt deliberately to solve several questions of great psychological interest, and this in a manner from which we may even yet learn a lesson.

We shall, as far as is consistent with the space at our disposal,

* "History of England from the Accession of James II.," vol. I. c. iii.

let Bulwer tell his own story. His works are now very rare,* and but few persons are familiar with them. Moreover Chaucer instructs us that—

> "Who so shall telle a tale after a man,
> He most reherse, as neighe as ever he can,
> Everich word, if it be in his charge,
> All speke he never so rudely and so large;
> Or else he must tellen his tale untrewe
> Or feinen thinges, or finden wordes newe."

To adopt the latter expedient would be to offer our readers an artificial for a real flower, to set before them mock-turtle when real was in the larder.

Bulwer's works are five in number, each being complete in itself, but each forming a portion of a wide scheme of observation and research. The two earliest published works—*Chirologia* and *Chironomia*—are found under the same cover, and with the following common title-page, which well expresses the nature of the books:—

"CHIROLOGIA: or the natural language of the HAND! Composed of the Speaking Motions and Discoursing Gestures thereof. Whereunto is added CHIRONOMIA: or the ART of MANUALL RHETORICKE. Consisting of the Natural Expressions, digested by Art in the HAND, as the chiefest Instrument of Eloquence, by HISTORICAL MANIFESTO'S, exemplified out of the Authentique Registers of Common Life and Civil Conversation. With TYPES or CHYROGRAMS: A long-wished for illustration of this Argument. By J. B. Gent. Philochirosophus. —*Manus membrum hominis loquacissimum.* LONDON. Printed by *Tho. Harper*, and are to be sold by *Henry Twyford* at his shop in Fleet-street. 1644."

The volume is dedicated by Bulwer to "his Heroique Friend Edward Goldsmith of Graies-Inne, Esqr.," and in the dedicatory epistle he writes:—

"—Affecting no Dedication that rises above the levell of *Friendship*, having intentionally consecrated all the issues of my recesse and leisure to certain select Friends; This book by *prescription* and *signiority* of acquaintance as by a *Prerogative*, and by a reciprocation of *love* for your affection to it, falls to your *Tuition*. I confess some other of my digested thoughts struggled for precedencie, claiming by the analogie of *Natures* usuall course, and the *head* would have had the priviledge of *primogeniture*. But it fell out in the contention somewhat like as in the case of *Tamar's* twins, where *Zarah* put forth his *Hand*, and the midwife said, *This is come out first*. However this *Chirosophie* or first fruits of my *Hand* be accepted abroad, having put forth my *Right Hand* in signe of amity to you, and for performance of promise, there remaines nothing (most noble *Chirophilus*) but that you take it between Yours in token of warranty and protection, as the tender offspring of one who is Your affectionate Friend."

* There is a complete series of his works in the library of the British Museum.

The preface of the book is addressed—"To the Candid and Ingenious Reader: This Copy of my Idea; or the Hint, Scope, and general Projection;"—which are thus quaintly set forth:—

"The consideration in generall, and at large of humane Nature, that great Light of Learning hath adjudged worthy to be emancipate and made a knowledge of it selfe. (*Franc. L. Verulam. Viscount St. Albans, de Augmen. Scient. l.* 4.) In which continent of humanity hee hath noted (as a maine deficiencie) one Province not to have been visited, and that is Gesture. Aristotle (*saith he*) ingeniose et solerter, corporis fabricam, dum quiescit, tractavit, eandem in noctis, nimirum gestus corporis, omisit, that is, he hath very ingeniously and diligently handled the *factures* of the Body, which are no less comprehensible by art, and of great use and advantage, as being no small part of civill prudence. For the lineaments of the body doe disclose the disposition and inclination of the minde in generall; but the motions doe not only so, but doe further disclose the present humour and state of the minde and will, for as the tongue speaketh to the eare, so *Gesture* speaketh to the eye, and therefore a number of such persons whose eyes doe dwell upon the faces and fashions of men, do well know the advantage of this observation, as being most part of their ability; neither can it bee denied but that it is a great discoverer of dissimulation, and great direction in businesse. For, after one manner almost we clappe our *hands* in joy, wring them in sorrow, advance them in prayer and admiration; shake our head in disdaine, wrinkle our forehead in dislike, crispe our nose in anger, blush in shame, and so for the most part of the most subtile motions. Taking (therefore) from hence my Hint, I shall attempt to advance in the scrutinie and march after the scattered glances, and touches of Antiquity, tracing them through most classical Authors, with intent to reduce them into one continued and intire History, propounding this form to myself, to handle *Gesture*, as the only speech and generall language of Humane Nature. For ballast to the subject, and to make the matter in *hand* more sollid and substantive, I shall annex consultations with Nature, affording a glosse of their causes: and for the further embellishing thereof, I shall inrich most points of expression with examples both of Sacred and profane Authority, more especially drawne from Poets and Historians, the only great Doctors in this point of Humane literature; wherein, by the way, I shall lay claime to all metaphors, proverbiall translations or usurpations, and all kinde of symbolicall Elegancies taken and borrowed from Gestures of the Body, with the depredations the subtiler Arts of Speech have made upon them for the advancement and exaltation of their particular inventions and designes. All these (together with the civill rites, and ceremonies customes and fashions of divers Nations in their nationall expressions by Gesture, with the personall properties and genuine habits of particular men) being but as so many severall lines that meet in an angle, and touch in this point; I intend to reduce and bring home to their fountaine and common parent the Body of man. Two Amphitheaters there are in the Body, whereon most of these patheticall subtilties are exhibited by Nature, in way of *discovery* or *impression*, proceeding either from the effect of sufferance, or the voluntary motions of the Minde, which effect those impressions on the parts which wee call the Speaking Motions, or Discovering Gestures, and naturall language of the body, to wit, the *Hand* and the *Head;* in answer whereof, I intend two receptacles of the observations, falling within the compasse of their particular Districts, under the generall Titles of *Chirologia* and *Cephalalogia*—The *naturall language of the Hand*, and, *The naturall language of the Head;* and these two comprise the best part of the expressions of *humane* Nature. *Chironomia*, or the *Rule of Hand* is adjoyned as the perfection and sublimation of *Chirologie*, as *Cephalelonomia*, or the *Rule of the Head*, is to appear with *Cephalelogia*, as being the gratification of all Cephalicall expressions, according to the Lawes of *Civill Prudence.* The personall or genuine expressions fall in with these. What I finde remarkable in the naturall expressions of the other parts, I shall refer to a generall Rendevouze, wherein I shall take a muster of the Postures and Gestures of the body in generall. All that I shall have to say more to the *Hand* in point of *Gestures*, is under the title of *Chirethnicologia*, or the *Nationall expression of the Hand.* This I account my left Hand. By this *Clavis* (I suppose) the *Intellectual Reader* will see that the work will be supplementall to Learning, and not of supererogation, New, and in regard of the generality of the Designe, never attempted by any, affording profitable hints to such ingenious spirits, who desire to understand the mysterious properties of so admirable and important a piece of themselves."

The psychical aspects of gesture and movement constitute then the theme which Bulwer discusses in the Chirologia and Chironomia, and it is interesting to observe that this theme was suggested to him by a passage in Bacon's *De augmentis Scientiarum* (first published in 1605).

In the Chirologia Bulwer works out a complete alphabetical and phraseological system of Manual expression, and shows the practicability of its application to several useful purposes.

The hand he holds is by no means a bad substitute for the tongue, for from the use made of gestures in carrying on trade with foreign nations, whose language is but imperfectly or not at all understood,—

"'Tis apparent, that there is no native law, or absolute necessity, that those thoughts which arise in our pregnant minde, must by mediation of our Tongue flow out in vocall extreame of words, unto which purpose we must attend the leisure of that inclosed instrument of speech. Since whatsoever is perceptible unto sense, and capable of a due and fitting difference, hath a natural competency to expresse the motives and affections of the minde, in whose labours, the *Hand*, which is a ready midwife, takes oftentimes the thoughts from the forestalled Tongue making a more quicke dispatch by gesture; so when the fancy hath once wrought upon the *Hand*, our conceptions are display'd and utter'd in the very movement of a thought. For the gesture of the *Hand* many times gives a hint of our intention, and speakes out a good part of our meaning, before our words, which accompany or follow it, can put themselves into a vocal posture to be understood. And as in the report of a Piece, the eye being the nimbler sense discernes the discharge before any intelligence by conduct of the vocall wave arrive at the eare; although the flash and report are twins born at the instant of the Pieces going off, so although Speech and Gesture are conceived together in the minde, yet the *Hand* first appearing in the delivery, anticipates the *Tongue*, in so much as many times the Tongue perceiving herself forestall'd, spares itself a laboûr, to prevent a needlesse tautologie. And if words ensue upon the gesture, their addition serves but as a comment for the fuller explication of the manuall Text of utterance; and implyes nothing over and above but a generall devoyre of the minde to be perfectly understood." (p. 4.)

If the details of Bulwer's system of manual expression are now regarded as somewhat cumbrous, yet it must be granted that they display a rare ingenuity and quick apprehension. The subjects of both the Chirologia and Chironomia are illustrated with neatly executed plates; and both works are preceded by curious allegorical frontispieces.

The laudatory verses which preface the Chirologia, manifest in a very curious manner the influence which Bacon's writings were, at the time when the work was published, beginning to exercise over literary men. One gentleman, tendering his rhythmical homage to Bulwer, writes:—

"Since the *Great Instauration* of the Arts
By *Verulamian Socrates*, whose parts
Advancéd *Learning* to a perfect state,
Thou art the first that from his *hints* must date,
For arts bemoan'd *defects* a new supply,
(The hardest Province in Humanitie).
Which doth in thy *Projections* ample spheare,
Another *Novum Organum* appeare.
And as we much unto Thy Hand doe owe
For *Augmentation*, some as farre shall goe
Another way, to shew their learned might,
While *Science*, Crescent-like, extends her light."

Another admirer writes:—

"Let *Bacon's* soule sleep sweet: the time is come
That *Gesture* shall no longer now be dumbe;
And Nature's silent motions shall advance
Above the Vocall Key of utterance:
There every *Digit* dictates, and doth reach
Unto our sense a mouth-excelling speech.
Arts Perfector! what *Babell* did denie
To Lips and Eare, t' hast given the Hand and Eye;
Hast reconciled the world, and its defect
Supply'd by one unmeaning Dialect."

A third, most attracted by the immediate practical applications of Bulwer's ideas, exclaims:—

"All that are deafe and dumbe may here recruite
Their language, and then blesse thee for the mute
Enlargement of thy Alphabets, whose briefe
Expresses gave their Minds a free reliefe.
And of this silent speech, Thy *Hand* doth shew
More to the world than ere it look'd to know.
He is (that does denie Thy *Hand* this right)
A Stoique and an Areopogite."

The title-page of the Chironomia runs as follows :—

"CHIRONOMIA : or the Art of *Manual Rhetorique*, with the *Canons*, *Lawes*, *Rites*, *Ordinances*, and Institutes of RHETORICIANS, both ancient and moderne, touching the artificiall managing of the HAND in speaking, whereby naturall GESTURES of the HAND, are made the Regulated accessories or faire spoken adjuncts of RHETORICALL Utterance. With TYPES, or CHIROGRAMS : A new illustration of this Argument.—By J. B. Philochirosophus.—*Ratio est Manus Intellectus; Rationis Oratio; Orationis Manus*. Scal. LONDON: Printed by Thos. Harper, and are to be sold by *Henry Twyford*, at his shop in Fleet-Street, 1644."

Although Bulwer simply gives the initials of his name on the title-pages of both the *Chirologia* and *Chironomia,* he signs his name in full at the termination of the dedicatory epistles contained in the works. After the publication of these works be became known as the *Chirosopher*, a cognomen which he adopted in the books he subsequently printed.

The publication of the *Chirologia* and *Chironomia* was followed, in 1648, by that of the *Philocophus*. The title-page of this work reads thus :—

"PHILOCOPHUS : or *the Deafe and Dumbe Man's Friend*. Exhibiting the Philosophicall verity of that subtile Art, which may enable one with an *observant Eie*, to *Heare* what any man speaks by the moving of his lips. Upon the same Ground, with the advantage of an Historical Exemplification, apparently proving, that a man borne Deafe and Dumbe, may be taught to *Heare* the sound of *words* with his *Eie*, and thence learne to speake with his Tongue.—By I. B. sirnamed the Chirosopher.—*Sic canimus Surdis—London*. Printed for *Humphrey Moseley*, and are to be sold at his shop in Paul's Church-yard, 1648."

This work is dedicated to "the Right Worp$^{ll}$ Sir *Edward Gostwicke*, of *Willington*, in the County of Bedford, Baronet; and M. *William Gostwicke*, his yongest Brother: and all other intelligent and ingenious Gentlemen, who as yet can neither heare nor speake. To be communicated unto them that can, and have acquaintance or alliance with any whom it may concern."

In the dedicatory epistle Bulwer writes :—

"—What though you cannot expresse your mindes in those verball contrivances of man's invention ; yet you want not *speech*, who have your *whole body*, for a *Tongue*, having a language more naturall and significant, which is common to you with us, to wit *gesture*, the generall and *universall language* of *Humane nature*, which when we would have our speech to have life and efficacy wee joyne in commission with our wordes, and when wee would speak with most state and gravity, we renounce wordes, and use *Nods* and other naturall signes alone. . . . .

"When coasting along the borders of *gesture*, and *voluntary motion*, I discovered a *community* among the *Senses*, and that there was in the continent of *Humanity* a *Terra incognita* of *Ocular audition;* a treasure reserved for these times, which had escaped their privy search, who guided by the illumination of their own endeavours had in *sudore vultus* ransackt the bosome of nature, wherein wisdome had hid it among other Arts and Sciences which have their foundation in Nature, and neither grow nor increase, but appeare when time and observation unlockt them unto us: Having well scanned this *magna naturæ*, I found it to be one of the subtlest pieces of Recondit learning, and that it bordered upon other avenues unto the braine, as *Orall* and *Dentall Audition*, of which we have discovered sufficient ground to raise a new Art upon, directing how to convey intelligable and articulate sounds another way to the braine than by the eare or eye; showing that a man may heare as well as speake with his mouth. Upon which and other unlooked-for discoveries, I began in idea, to conceive the modell of a *New Academie*, which might be erected in favour of those who are in your condition, to wit, originally deafe and dumbe."

Thos. Diconson, Esq., of the Middle Temple, who acts as chief chorus to Bulwer's writings, has so aptly expressed the object and tendency of the *Philocophus*, in certain laudatory verses, prefixed to the book, that we should err if we did not quote them.

"Rejoice, you Deafe and Dumbe, your Armes extend
T' embrace th' inventive goodnesse of a Friend!
Who heere intends, for your relief to Found
An Academie, on NATURE's highest ground:
Wherein He doth strange mysteries unlocke,
How all the Sences have one common Stocke,
Showes how indulgent Nature for each sence
Wanting, allows a double recompence.
How she translates a sence, transplants an Eare
Into the Eye, and makes the Optiques heare.
Inoculates an Eare with sight; whereby
It shall performe the office of an Eie.
Presents rich odours Tasted, viands Smelt,
And Sound and Light in a strange maner felt.
The sences (Art's new Master-piece) are taught
T' exchange their objects by a new-found thought.
The Deafe and Dumbe get Hearing Eies, which breake
Their Barre of Silence, and thence learne to speake.
Words may be seene or heard: w' are at our choyce
For to give Eare or Eie unto a Voyce.
Where men by their transposed sences gaine,
This Anagramme of Art and Nature's plaine."

Bulwer thus sums up the "Hints and Notions" he has expressed in the *Philocophus,* which "more directly concerne Deafe and Dumbe men:"—

"That men born *Deafe* and *Dumbe* have a kind of significant speech and naturall language; and what that is.

"Wherefore it is that *Deafe* and *Dumbe* men can expresse themselves so lively by signes.

"That all *Deafe* and *Dumbe* men seeme to have an earnest desire to unfold their lips to speak, as if they accounted their *Dumbness* their greatness unhappiness.

"That a man born *Deafe* and *Dumbe* may be taught to *heare sounds or words with his eyes.*

["That *articulate speech* doth not necessarily assist the audible sound of the voyce, but may consist without it, and so consequently be seen as well as heard.—c. xiv.]

"The strangenesse of that expression abated and qualified, by proving a community among the sences, and their mutuall exchanging of objects.

"And hearing to be nothing else but the due perception of motion."

We now come to the rarest and, in a psychological point of view, perhaps the most interesting of Bulwer's works, the *Pathomyotomia.* In this work we see still more thoroughly worked out the ideas which had governed Bulwer's previous writings. Moreover, from it we learn how clearly he apprehended the importance of these ideas as clues to a wide, and at that time, almost undisturbed field of research. The title-page of this, Bulwer's fourth published work, is as follows:—

"Pathomyotomia, or a Dissection of the significative *Muscles* of the Affections of the Mind. Being an Essay to a new Method of observing the most Important movings of the *Muscles* of the Head, as they are the nearest and Immediate Organs of the Voluntarie or Impetuous motions of the Mind. With the Proposall of a New Nomenclature of the *Muscles.*—By J. B., Sirnamed the *Chirosopher.* —*Augebitur Scientia.*—London, Printed by W. W. for Humphrey Moseley, and are to be sold at his shop at the *Princes Armes* in St. *Pauls* Churchyard. 1649."

The author dedicates this work to "his loving Father, Mr. Thomas Bulwer," writing (and this dedicatory epistle contains a curious piece of contemporary medical history),—

"It hath been a laudable Custome with Persons of eminent degree to descend to honour their Sonnes with directing Books unto them; the reciprocation of which affectionate Complement is a duty well-becomming a Son; moved (therefore) with a certaine Filiall Decency, I made choice to dedicate this Book unto you in regard the Argument of it is Provinciall to Physick, wherein your experience hath crowned your Profession, having ever been *Fortunatus in Praxi.* You shall find in it that which I use to call the Clock-work of the Head, or the Springs and inward Contrivance of Instruments of all our outward motions, which give motion and regulate the Dyall of the Affections, which Nature hath placed in the Face of Man; Being a New light, and the first Irradiation which ever appeared through the Dissections of a Corporeall Phylosophy. Could the Times have afforded it, it had come to your hands illustrated with the Ornamental Demonstrations of many Figures prepared for it; but indeed the Stationers cautionary prudence met with an indisposition in me; for I thought that in such new and unexpected matters too great a splendor might possibly have dazled. I confess I have met with little encouragement in this Designe, for all the Physicians and Anatomists that I have hinted it unto have held it scarce fecible, Doctor *Wright* Junior onely excepted, with whom having interchangeably communicated Intellectual Affaires, He shewing me the hint of his grand undertaking, which was *Anatomia Comparata*, that great Defect in Anatomy noted by my L. *Bacon* in his book *De Augmentis Scientiarum*, in returne whereof I having first told him of an Atchievement of mine in this Art, which I called *Vox Corporis*, or the Moral Anatomy of the Body; I acquainted him also with this Essay, whose apprehension I found so well possessed with the gallantry (as he was pleased to speak) and the possibility thereof, that he promised me (to testifie his approbation,) he would commend it in his first publique Lecture of Anatomy in the College; a day much expected by those who had took notice of the most eminent and Divine Impulsions of his Anatomique Genius. But prevented by his much lamented Death, what entertainment this Essay shall meet with among the Sect of Corporeal Philosophers, (having bin so infortunately deprived of the advantage of such a Recommendation,) must be left to the Fate of Books. However, I hope it shall find acceptance with you, and be received as an evidence of the proofe of that Education you bestowed on me, and of my Duty."

Bulwer next proceeds to unfold "The Scope and Use of the Essay for some previous satisfaction to the Intelligent Readers. More especially Physicians and Masters in Anatomia, whose Candor and Indulgence on this Essay is most properly desired." This is the cream of the book, and together with the fragments we have quoted from the *Chirologia, Chironomia,* and *Philocophus,* will enable our readers to form a tolerably accurate judgment of Bulwer's habits of thought. He writes:—

"Having resolved to trace the Discoursing Actions of the Head to their Spring and Principle upon which their outward significations depend; when I had passed the superficial parts, and digged a little more than skin-deepe into the Minerall of Cephalicall Motion, I came to the *Muscles,* the instruments of voluntary motion; or the instruments of those motions that are done by an earnest affection, that is, from an inward principle. The effects of whose moving significantly appeare in the parts moved; when by an arbitrary motion we freely reject or embrace things understood (not with our mind only, but with our mind and body both). Here I made a stand; and began curiously to enquire and hunt after all the Anatomists both Ancient and Moderne that had writ of the *Muscles*, and the motions of the Head, as well to satisfie my self as to crave in ayde of them: and having had a view of as many as I could heare of and conveniently procure, and observed their severall views and methodicall variations no way answering my expectations; An emergent thought suggested to my imagination a notable Defect, hitherto undiscerned in that Art which of late hath attained unto a great perfection, which cast me into an extasie of admiration at so strange a Preterition, that among the Conscript Fathers of Anatomy there hath not been any one who, *Datâ operâ*, had undertaken a generall survey and Cognomination of the muscles of the Body, as they are the necessary Instruments of all those motions of the Mind, which are apparently expressed and made manifest by the effect of their use and movings in all the parts of the Body, although more Emphatically, by those operations they have in the Head and the most remarkable parts thereof. *Galen*, in that excellent Commentary, *De Motu Musculorum*, wherein he went beyond himselfe, and shewed the greatest miracle of his wit, a Book which all Anatomists kisse with reverence, as containing the Oracles of Myologie, doth not so much as glance at it, but under the generall notion of voluntary and arbitrary motion; and in his Anatomicall Administrations wherein he abundantly prosecutes these motions, and glories to have found out many which were hid and unknown to the Physicians that were before his time, and where he teacheth a method whereby every single motion may be found out; Nor in his Dissection of Muscles not a word, scarce, pointing to this Intention, not naming many, but only numerically, not three with an imaginable reference to any Emphaticall motion of the Mind. And all the Nomenclators since his time, who have undertaken to play with new names, which for memory, brevity of speech, and perspicuity of the thing, they have imposed upon the Muscles, have omitted the due regard proper to the Spirit and Life of their mentall significations: whereas the Denomination had been better from the Nature and energeticall property of the Muscle, which should by that Rule have been taken from the more extant and patheticall representation of those parts they actuate, and by which they exhibite their Organicall significations. More strange yet, that no Artists should have made this the Subject of their Orations, but should have all to this day, either turned their discourse to the structure onely of the Humane Fabrique, the perfecfections or Symetry of the Body, or the excellency and antiquity of the Anatomique Art, or the *Encomiums* of the Antient and Moderne Anatomists: whereas nothing could have set a greater glosse upon the Art, or have bin more glorious and honourable, than together with their Dissections, to have enriched their discourse with a relation of the Essence, Regiment, and proprieties of the Soule, whose well-strung instrument the Body was; Dr. *Floud* being the first that in his peroration (when he was Prælector of Anatomy in the College of Physicians in *London* Anno 1620) exhibited such a kind of Method, together with an Explanation of his Reason, and an Example thereof; which Forme he did not magisterially propound unto them, but to declare that the Subject of an *Anatomicall prœludium* ought to be the Internall and spirituall man, which is rather to be dissected with living words, than any knife how sharpe soever, and so consequently to be discovered and explained by a style of discourse. The field of which subject as it is more ample and spacious than the rest; So the Studious in Anatomy shall never find it barren, but most fruitfull; So that every one herein may hit of much variety of invention. If then a Prologue onely of this nature is held so convenient by so great an Artist, how much more advantageous and delightful would a discourse interwoven throughout the Dissection! Finding (therefore) that neither the great Parents of Physick, nor their Learned Off-spring had pathologized the Muscles, and thence bestowed significant names upon the most remarkable of them; I resolved to attempt the Designe, so to take away the blemish which hath fallen upon the Art by the slovenly and careless Denomination of some of them, and the six-footed Barbarismes of those Greeke Conjuring names which are fit only for the bombastical Anatomy of *Paracelsus;* wherein I was encouraged by observing that half a dozen of Muscles named according to our new intended modell, or the Species of their most significant motion; and seeme to have been stumbled upon by the way of sport, or a Rhetoricall Chance-Medley of wit; appeare so wonderfully pleasing to our moderne and most ingenious Anatomists, that they are still borrowing from one another those patheticall Apellations, or as *Riolanus* calls them, *Elegantissima nomina,* as if they were much affected with the felicity of that Pen from whence they first distilled, *quæ omne tulit punctum,* for Elegancy, Memory, Brevity, and Perspicuity. 'Tis true many have exercised their pens in discourses of the muscles: But an exact Description of the Discoursing Motions of the Muscles none of the Great Professors of Anatomy have so much as thought on; whereas the facility, utility, and delightsomenesse of such motions might have invited many; for, what is more easie than to discerne the parts manifested to Sense, and the fidelity of an Ocular assurance? that are so subject to our touch, that in the semblance of those motions wrought in the parts by the endeavour of the Muscles, we may not only see, but as it were feele and touch the very inward motions of the Mind; if you aske what delight will hence acrew to the understanding? What is so delightfull as to know by what kind of movings those varying motions and expressions of the Head and Face are performed? What Muscle doth accomplish this or that speaking motion? To observe the scheme or outward figure of each Affection in the Countenance? That is the situation of each in its Motion, as it is drawn by the Muscles, and to read their significations couched in their names? So that observing these accidents of the Head and Face, the Types and representations of the Affections which are accidents of the Mind, according to the nature of Correlatives, we may find out one by the other. And though it be but Negative ignorance not to be skilled in such matters, and so may be thought a needlesse Nicety or over-curious Inquisition to know every Muscle of our Head and Face: Yet certainly it cannot but be some disparagement to one that pretends to any ingenuous Education or Reading, to be as a meere Puppet or Mathematicall motion, and not to understand why, or after what manner, the Muscles of his Head move in obedience to the command of his Will; and so to have no better a Head-piece than that, which counterfeiting the naturall motions of Speech, uttered its mind to *Thomas Aquine,* and the learned Frier *Bacon.* And who I pray you that is well versed in Philosophy, does affect to behold the cold effects of common Actions, without a Discourse of their Causes and intrinsicall Agents—the Soule and the Muscles ? Since that is familiar to Sense, and so by consequence to Beasts,—But this is subjected to the Intellect, to wit, the Internall Principle of man, wherefore we will think it a thing worthy to be corrected with the whip of Ignorance, if any rashly plunge himself into the Muscular Sea of corporal Anatomy, or of the outward man, without any mention of the Internall man, since the Soule only is the Opifex of all the movings of the Muscles, whose invisible Acts are made manifest by their operations in those parts into which they are inserted. Not that any perfection or exact knowledge of this nature can be acquired; since the wisedome of the Creator in the fearefull and wonderfull structure of the Head is not yet fully found out, although it has been sought after by illustrious men with much piety and Diligence: and therefore that which is most probable, and has the countenance of Authority, must passe for truth. To those also that shall hereafter Physically and Ethically handle the Doctrine of humane Affections, this may serve as a *Mercurius Ethicus*, to give intelligence to all *Athenian Pathologists*, of the motions of the Muscles which beare the greatest sway in matter of Affection: whereas heretofore Pathology hath beene confined, as it were, to *Aristotle's* Muscle, to wit that principle of inbred Heate, or ever movable substance of Spirit and blood, which seemes to frame the severall images of all the affections of the Mind; and has had little or no entercourse with the Muscles of the Affections; whereby she has been deprived of a great part of this ornament whereof shee is capable. But perchance the modernes have bin frighted with the difficulty of such Designe, as supposing such a *Muscular Philosophie* not fecible or reduceable into an Art: or else if it ever came into their Heades, they thought it a kind of impudence after *Galen* that glorious light of Anatomy, to endeavour any thing in this kind. Yet *Galen* in his Booke *De Motu Musculorum* seemes to have given any one a faire occasion of daring, where he writes, Whereas we have partly found out many things, and partly also intend diligently to make a thorow search after other things; and some other may find out what is wanting. With his leave therefore, I shall endeavour by a light Essay, to take notice of the figure and signatures of those Muscles that belong unto the Head, and are the Authors of the speaking motions thereof, and of the Superficiall parts comprised in it, by the way, raising Allegoricall inferences from them, and adapting and imposing new names upon them according to their Physiognomical significations, which shall be as the Keyes of their important actions. Describing the rising and insertion, together with the fibres which modify the Determinate actions of each Muscle that I find, instrumentall, adjutant, or any way concurring to the expedition of any remarkable gesture of the Head or Face: So ordering the matter, that occasionally, most, if not all the mysteries of voluntary motion shall be brought in, at least in such a manner, as shall be more than sufficient to lay a firme foundation to our virgin Philosophie of Gesture, and to serve my turne for the present occasion. And because none that hitherto have treated of the moving of the Muscles, have driven after this Scope of their significations; I shall name the authors by whose light I walke, and upon whose Bowle I clap the Bias of the Affections, neither my Margin, nor the nature of an Essay admitting any more criticall formalities of quotation. I am not ignorant that such daring attempts and undertakings are very obnoxious to envy, and apt to fall under the censure of Arrogancy and ostentation, imputations I have no reason to feare, since I arrogate, not to my selfe by the conduct of my owne light, to have found any new or great thing to add to the Doctrine of Muscular motion, to which (to speak the truth) I thinke there cannot much be added: neither am I so conceited of these animadversions, as to hope they should be admitted into the schoole of Anatomy, and straightwise be made Canonical; for, to fit a Novelty of this nature for such an admission, would require a whole College, or rather a nationall Synod of Anatomists to consult about it; my single Phantsie being not therefore *par negotio*, I have adventurd far with little strength and lesse encouragement to recommend the Designe to men of stronger Brains and publique Spirits. I think I may with modesty suppose, that I have sprung a new veine, and say that I was enforced to dig my way through, and out of much Oare and Drosse, to refine what was fit for my purpose before I could come to ransack this Secret and undiscover'd treasury of the Muscles; or to cast the old metall of their matter into a new mold, to make it more illustrious by conjoyning it with the inward motions of the minde, which set a representative shape and glosse upon the outward motions of those parts which are moved by the Muscles. If they are contented to allow me to have bin the first that by Art endeavoured to linke the Muscles and the Affections together in a new Parthomyogainia; or at least to have published the Banes between *Myologus* and *Pathology*, that any Physiologicall Handfaster that can marry them stronger together, might doe it if he pleas'd: I aske no more: as for the rest, *Veniam pro laude peto.* And if the *Scorbutick* wits of this Age, who preferre an idle Head before an active, should bee loath to afford me that, I can easily comfort my selfe with that of *Cremutius in Tacitus*, *Suum Cuique posteritas rependit, nec deerunt, si Damnatio, ingruit, qui mei meminerint.*"

The excellent and far-reaching philosophy of the preceding observations of Bulwer must certainly commend itself to our readers. It is perhaps not too much to say that, neither Lavater in his great work on *Physiognomy*, nor Bell in his *Anatomy of Expression*, manifested that lofty conception of the subject with which each dealt, that Bulwer did. With him Physiognomy and Expression, even when the latter was restricted to its connexion with the Fine Arts, were the natural offshoots of a wide-extending idea so fertile in its application and results, that the latter are still far from being exhausted. Doubtless, from the imperfect state of anatomy in Bulwer's time, he, in elaborating and applying his idea, fell almost as far short of success as Bacon did, when that great philosopher endeavoured to practise the method he so admirably taught. Yet, we think, that had an idea as pregnant and as philosophical as the one which guided Bulwer, influenced Lavater and Bell, when, with pen and pencil, they sought to give definiteness to our knowledge of the outward manifestations of the mind, their works would have possessed a higher and perhaps more abiding interest than they now possess.

The last, most curious, and most generally known of Bulwer's works is, the *Artificial Changling*, the title-page of which runs thus:—

"ANTHROPOMETAMORPHOSIS; Man Transformed: or the ARTIFICIALL CHANGLING, Historically presented in the mad and cruell Gallantry, foolish Bravery, ridiculous Beauty, filthy Finesse, and loathsome Lovelinesse of most NATIONS, fashioning and altering their Bodies from the mould intended by NATURE. With a Vindication of the Regular Beauty and Honesty of NATURE. And an Appendix of the Pedigree of the ENGLISH GALLANT. By J. B., Sirnamed the Chirosopher.—*In nova fert animus, mutatas dicere formas.*—London, Printed for *J. Hardesty*, at the Black-spread-Eagle in Duck-Lane, 1650."

This, the first edition of the work, is in duodecimo, and contains 263 pages of text, exclusive of prefatory matter and tables of contents, these not being paged. The book has a folding frontispiece exhibiting, on the one side, various heads illustrative of the text; on the other, a portrait of the author. It would not be advisable to attempt to describe Bulwer's physiognomy from this counterfeit presentment of him, and it is doubtful even whether the face which is represented, is that of a man of mid or in the decline of life.

Another edition of the *Anthropometamorphosis* was published in 1653. This is a small quarto work, and the title-page contains, after the words "intended by Nature," the following addition:—" With figures of these Transfigurations. To which artificiall and affected Deformities are added all the Native and Nationall Monstrosities that have appeared to disfigure the Humane Fabrick." The authorship and printer of this edition are thus stated:—" Scripsit J. B. Cognomento Chirosophus, M.D.—London, Printed by William Hunt, Anno Domini 1653." The book contains 569 pages, in addition to the introductory matter and table of contents. The title-page is preceded by a most singular frontispiece, and the cuts dispersed throughout the book are very curious, and as roughly executed as curious. W. T. Turnbull, Esq., of the Record Office, the learned editor of the lately published Metrical History of Scotland, has in project a republication of the last edition of the *Anthropometamorphosis*, with fac-similes of the cuts. If, as we hope, this project should be carried out by our worthy friend, we can promise our readers (such of them at least as are bibliographically inclined) a rare treat.

The *Artificial Changling* is dedicated to Thos. Diconson, Esq., and in the dedicatory epistle Bulwer writes:—

"Friend, the Heroique Disease of writing hath (as you well know) long seized upon me, this being the Fifth Publique *Paroxisme* I have had thereof. It hath been ever the humour of my *Genius* to put me upon untrodden pathes, and to make up aggregate Bodies of very scarce and wide dispersed notions; which had been more easie for the Faculty of my weak Body, had I had a signality of spirit to summon Democriticall Atomes to conglobate into an intellectual Forme; or that *Mercury* had been so propitious a Lord of the Ascendant in my Nativity, as he was *Amphion's*, and bestowed some *Orpharion* upon me, with whose sound I might have attracted notions, and made them come dancing to the Construction of a Book. What I here present you with, is an Enditement framed against most nations under the Sun; whereby they are *arraigned* at the Tribunal of Nature, as guilty of High-treason, in Abasing, Counterfeiting, Defacing, and Clipping her Coine, instampt with her Image and Superscription on the Body of Man. . . . . . . .

"I doubt not you will soone discerne the prepense malice of Satan in it; tempting mankind to a corporall Apostacy from himself, as if in an Apish despight of the glory of Man's Creation, that divine consultation, *Faciamus hominem*, Let *us* make man according to our Image; He would have his *Defaciamus hominem*, Let us deface man according to our likeness; insomuch as that of the *Psalmist*, *I am fearfully and wonderfully made*, might be ironically applyed to man in this *abusive Transformation*."

Bulwer explains that this portion of his "Corporall Philosophy," is an "Historicall Tract of the Use and Abuse of Parts," and is intended to teach the foolish perversity of man in running into error; and he would have the book to "serve as a Glasse for the pernitiously-affected Gallants of our own time to looke in, and see the deformity of their Mindes and their Pedigree and Alliance; who practise such phantasticall emendations of nature, as dishonour her, and apparently show that they glory in their shame. And that men descending into themselves, may know themselves to *be men not beasts*, and learne to order the August Domicil of man reverently to the health of the Body and honour of the Soule."

The subject of the "*Anthropometamorphosis* may have been suggested to Bulwer (as that of the *Chirologia* was), by a passage in the *Advancement of Learning*.

Bacon, in that work, teaches us that "the knowledge that concerneth man's body is divided as the good of man's body is divided." Now, "the good of man's body is of four kinds, health, beauty, strength, and pleasure; so the knowledges are medicine, or art of cure: art of decoration which is called cosmetique; art of activity, which is called athletique; and art voluptuary, which Tacitus truly calleth 'eruditus luxus.'" Further we are taught that *Cosmetique* "hath parts civil and parts effeminate: for cleanness of body was ever esteemed to proceed from a due reverence to God, to society, and to ourselves. As for artificial decoration, it is well worthy of the deficiencies which it hath; being neither fine enough to deceive, nor handsome to use nor wholesome to please."

This last sentence may possibly have prompted the "Artificiall Changling." Bulwer, however, in dealing with his subject displays all the originality of a thoroughly active and thoughtful mind. The outrageous extravagancies of dress and personal decoration which had been in vogue previous to the fall of the monarchy, were favourite subjects of the moralists and satirists of Bulwer's day; and an admirable illustration of their method of dealing with the matter is to be readily found in the learned wrath which Burton lavishes upon *Artificial Allurements*, in his *Anatomy of Melancholy*.* But Bulwer endeavours to construct a comparative view of the methods of artificial decoration as well as of the artificially produced deformities of mankind generally, and so doing, maugre the imperfect materials at his command, he not only achieves the main,—the moral object which he aimed at, but, by his method and arrangement, he breaks the ground for a psychical comparison of different races of men.

Of course we, in our advanced wisdom, are provoked to much merriment by the wonderful recitals which are plenteously scattered throughout this book. For example, there is the familiar instance quoted by Dr. Nash in his notes upon Hudibras,† and familiar because so quoted. Bulwer, in the chapter on "Tailed Nations, Breech Fashions, and abusers of that part," relates, among other accounts of individuals possessed of a caudal appendage, a story told to him by "an honest young man of Captain Morris Company, in Lieutenant General Ireton's regiment, that at Cashell, in the County of Tipperary, in the province of Munster, in Carrick Patrick Church, seated on a hill or rock, stormed by Lord Inchequine, and where there were near seven hundred put to the sword, and none saved but the Mayor's wife and his Son; there were found among the slaine of the Irish, when they were stripped, divers that had Tailes neare a quarter of a yard long; the Relator being very diffident of the truth of the story, after enquiry, was ensured of the certainty thereof by forty soldiers that testified upon their oaths that they were eye-witnesses, being present at the Action."

This belongs to the category of errors which, as Bacon tells us, can be corrected by time alone. We can laugh at such credulous mistakes now, and can understand how, even when first

* Pt. III., sec. 2, mem. 3, sub-sec. 3.

† Pt. II. c. i. l. 742.

vended, they did not fail to come under the lash of shrewd men; but we cannot yet afford to lay too much stress upon the surpassing acuteness of our own days. There is always one or more of the descendants of Autolycus roaming about shouting forth some wondrous story "with five justices' hands at it, and witnesses more than his pack will hold;" always many children of Dorcas and the Clown ready to swallow with avidity the numerously attested tale. But when the children's part in this act of the drama is played by the Emperor and Empress of a great nation, as is reported of their Imperial Majesties of France in certain "spirit-rapping" exhibitions recently said to have been enacted before them, our laugh at John Bulwer's credulousness, as we affect to consider it, degenerates into a very melancholy quaver.

At the termination of the *Anthropometamorphosis*, Bulwer gives a list of the works which he had published (all of which we have now noted); also of the works which he had "accomplished," and which he might be induced "hereafter to communicate." That hereafter does not appear to have ever arrived, but it is proper, if we would obtain a right understanding of the man, to know the titles of the works which we have unfortunately lost. It may be well also to remark that the list of accomplished but not communicated works appended to the edition of the *Anthropometamorphosis* in 1653 contains two titles more than the list appended to the edition of 1644. The list of 1653 is as follows:—

"Chirethnicalogia: or, The Nationall Expressions of the Hand.

"Cephalelogia: or The Naturall Language of the Head, being an Extract of the most noble and Practical Notions of Physiognomy.

"Cephalenomia: or the Art of Cephalicall Rhetorick.

"Vox Corporis: or the Morall Anatomy of the Body.

"*The Academy of the Deafe and Dumbe:* Being the manner of Operation to bring those who are so borne, to heare the sound of Wordes with their Eyes, and thence to learn to speake with their Tongues.

"Vultispex Criticus: seu *Physiognomia Medici.*

"Glossiatrus: Tractatus de removendis Loquelæ Impedimentis.

"Otiatrus: Tractatus de removendis Auditionis Impedimentis."

This list, then, was alone needed (if the intimations given in the prefaces to his books had not sufficed) to show how completely Bulwer had apprehended, worked out, and applied to practical consequences of unusual importance, the play of the mind in gesture and movement. This list, also, completes our justification in claiming for Bulwer a notable position as a medical psychologist, and a loftier standing as a man of science than it has been customary to yield to him.

Bulwer deserves a very honourable place in the history of English, and, indeed, of European medicine. He first of all, apparently, reasoned out on thoroughly scientific grounds, the practicability of instructing deaf-mutes. He did this, certainly, at a time when Bonet's success in educating deaf-mutes in Spain was becoming known in England, and when there were other workers in the field besides himself. Indeed, a sentence in the title-page of the *Chirologia*—to wit, that the Types or Chirograms were "a long-wished for illustration of this argument"—would lead to the supposition that the subject of the book was one which had excited some attention previous to the publication of Bulwer's investigations. To others, so far as we can now ascertain, belongs the honour of first showing experimentally, in this country, the practicability of teaching the deaf and dumb. But this in no wise detracts from the peculiar merit of Bulwer; and even now we cannot but admire the effective manner in which he paved the way for and facilitated the labours of contemporary and subsequent workers in the same field. Bulwer, however, saw that the culture of deaf-mutes was but one of several important practical applications of the psychical principle from which he started; and it is the clear and full apprehension of the vitality of this principle, so quaintly set forth in the prefaces to his works, which, we conceive, constitutes his highest and best claims as a scientific man and medical psychologist.

Bulwer's works, as we have already intimated, possess the additional and collateral interest of being a charming illustration of the earlier and direct effects of Bacon's writings and method, on thoughtful literary and scientific men in this country. Bulwer derived his inspiration from Bacon, and that he made a most excellent use of this inspiration in the then unsatisfactory state of biological science, will hardly be denied.

What Bulwer chiefly deserves to be remembered and honoured for has been so well recited in the *Pathomyotomia*, by his principal chorus, Thos. Diconson, Esq., *Med. Templ.*, that we shall permit this gentleman again to recount the merits of his friend in resounding verse. Nay, the verses which we are about to quote are worth reading as verses, if for their quaintness merely, and they are by no means a bad example, and might serve as a capital type of eulogistic poesy. Dedications of books are once more becoming the fashion, and who knows but that laudatory verses may again come into requisition?

"*To the daring* ADVANCER *of all* SOMATICALL SCIENCE, *his Selected Friend, on his* PATHOMYOTOMIA.

"Thou Grand Adventurer, wits *Magellan*,
To whom our Microcosme or Isle of Man,
By thy all searching Pen 's so thoroughly scand,
There's now no part in us an unknown land;

How thriving is thy head in new Designes,
To bring home, not the minerall, but the mind.
This Pathologicall Anatomy,
Deare Friend, hath wound our admiration high.
A strange Essay, indeed, that dares to trace,
All the rare Springs and Wards that move a face;
To make Anatomy by muscles wind
The swiftest motions of the winged Mind,
Nature's high piece of Clockeworke this you call,
Reason the Spring winds up, the Muscles all,
Like wheels move this or that way, swift or sloe,
As the Affections' weight doth make them go.
All the Soules motion's seen, the Head and Face,
Discovering all, as through a Cristall Case;
Here the Affections keep an Open Marte,
By Patent seal'd by thy Cephalick Art.
The Itchnographie of the Art do's smile,
A promis on us, of some stately pile,
And puts us in good hope abroad to see
That Masterpiece of Physiognomie,
Thy Magisteriall Quintessence of Bookes,
Or extract Scientificall of lookes.
Then that whereby as a Face-Prophet shown,
Thou know'st the Affections' are the Bodye's own,
Whence subtiley thou'rt wont to ken and trace,
The Criticall Disease-discovering Face,
That though the humors bedded are within,
Yet thou canst track their footsteps in the skin.
Strange secrecy of Art and mysticall,
To cast our Faces as an Urinall;
Nay, by a stranger's Face well copyed out,
For to pronounce by Art He hath the Goute.
Such are thy common Augries; we may
Sure trust thy skill, that doth such beames display.
The last Yeare stil'd you Deafe and Dumbe man's friend,
Now Thy Design more deeper doth descend.
I see Thy knowledge and invention flowes
As far in man as Sense and Motion goes.
Then take the Chair, where maist Thou Doctor sit,
Command our health's, as Thou hast done our wits.
Lure down thy soaring Truths, solve every doubt,
And by convincing practice make them out
To Faith-bound Sceptiques, who count nothing good
Till flat experience make it understood.
Now to the Art-forsaken Deafe dispence
Thy skill as Aurist to restore their sense.
Wert thou once known Surdasters would come on
To court Thee for Thy Autocousticon.
Next as a Linguist teach the dumb to breake,
Or pick the Padlock lets his Lips to speake.

Ransom each captiv'd tongue, weak speech improve,
And the impediments thereof remove.
Then as a Motist by this healing light,
Set all our Heads' depraved motions right.
And may success attend, while swelling Fame
Fills up thy Sailes with an All-healing name."

---

### Art. III.—THE INDEPENDENCE OF THE SOUL:

